# Emergency services utilization in Jakarta (Indonesia): a cross-sectional study of patients attending hospital emergency departments

**DOI:** 10.1186/s12913-022-08061-8

**Published:** 2022-05-13

**Authors:** Syaribah Noor Brice, Justin J. Boutilier, Daniel Gartner, Paul Harper, Vincent Knight, Jen Lloyd, Aryono Djuned Pusponegoro, Asti Puspita Rini, Jonathan Turnbull-Ross, Mark Tuson

**Affiliations:** 1 Cardiff School of Mathematics, Cardiff University, Senghennydd Road, Cardiff, CF24 4AG UK; 2 Department of Industrial and Systems Engineering, University of Wisconsin – Madison, 1513 University Avenue, Madison, WI 53706 USA; 3 Welsh Ambulance Services NHS Trust, Vantage Point House, Ty Coch Way, Cwmbran, NP44 7HF UK; 4 118 Emergency Ambulance Service Foundation, Jl. Pahlawan Raya No. 50, Rempoa, Ciputat Timur, Kota Tangerang Selatan, Banten 15412 Indonesia; 5 Welsh Ambulance Services NHS Trust, Business Park, Ty Elwy, Ffordd Richard Davies, St Asaph, LL17 0LJ UK

**Keywords:** Pre-hospital, Emergency medical services, Ambulance, Indonesia, Low- and middle-income countries

## Abstract

**Background:**

Pre-hospital and emergency services in Indonesia are still developing. Despite recent improvements in the Indonesian healthcare system, issues with the provision of pre-hospital and emergency services persist. The demand for pre-hospital and emergency services has not been the subject of previous research and, therefore, has not been fully understood. Our research explored the utilization of emergency medical services by patients attending hospital emergency departments in Jakarta, Indonesia.

**Methods:**

The study used a cross-sectional survey design involving five general hospitals (four government-funded and one private). Each patient’s demographic profile, medical conditions, time to treatment, and mode of transport to reach the hospital were analysed using descriptive statistics.

**Results:**

A total of 1964 (62%) patients were surveyed. The median age of patients was 44 years with an interquartile range (IQR) of 26 to 58 years. Life-threatening conditions such as trauma and cardiovascular disease were found in 8.6 and 6.6% of patients, respectively. The majority of patients with trauma travelled to the hospital using a motorcycle or car (59.8%). An ambulance was used by only 9.3% of all patients and 38% of patients reported that they were not aware of the availability of ambulances. Ambulance response time was longer as compared to other modes of transportation (median: 24 minutes and IQR: 12 to 54 minutes). The longest time to treatment was experienced by patients with neurological disease, with a median time of 120 minutes (IQR: 78 to 270 minutes). Patients who used ambulances incurred higher costs as compared to those patients who did not use ambulances.

**Conclusion:**

The low utilization of emergency ambulances in Jakarta could be contributed to patients’ lack of awareness of medical symptoms and the existence of ambulance services, and patients’ disinclination to use ambulances due to high costs and long response times. The emergency ambulance services can be improved by increasing population awareness on symptoms that warrant the use of ambulances and reducing the cost burden related to ambulance use.

**Supplementary Information:**

The online version contains supplementary material available at 10.1186/s12913-022-08061-8.

## Background

Emergency medical services (EMS) are a critical part of any healthcare system and provide emergency care in primarily pre-hospital settings. The provision of pre-hospital emergency services in low- and middle-income countries (LMICs) has been historically characterised by inadequate resourcing both financially and operationally (e.g., in terms of staff) [1]. Other challenges associated with EMS in LMICs include decentralized services, fragmented data collection strategies, and high variability in the quality and consistency of collected data [2, 3]. The combination of inadequate infrastructure and culture differences associated with healthcare-seeking behaviour, communication, and coordination represent significant barriers to EMS access in these countries [4].

In Indonesia, the healthcare system is undergoing significant changes supported by the government across many areas including emergency care in pre-hospital settings [5]. For example, Jakarta (the capital city) has seen a large increase in the number of government ambulances and the establishment of an emergency call centre [6]. However, significant challenges facing pre-hospital care in Indonesia remain including a lack of ambulance availability in many areas, political and economic uncertainty, and low public awareness of the existing EMS system and emergency care options [6]. Furthermore, the predominant fatalistic culture in Indonesia, where people believe that their destiny is pre-determined, is known to influence individual behaviour in relation to seeking medical help [7].

Approximately 32.4% of the population in Indonesia reported having health concerns in 2019 [8] and 13.8% of the population in Jakarta has significant health problems [9]. In particular, the fraction of total deaths caused by time-sensitive emergencies (e.g., trauma) was significantly higher than other LMICs in the Asia Pacific region. For example, around 19.4% of total deaths in Indonesia were caused by stroke, as compared to 10.5 and 9.8% for other high-income and LMICs in Asia Pacific, respectively [10]. Moreover, Indonesia experienced twice as many deaths from trauma (e.g., car accidents) as compared to high-income countries in the Asia Pacific region [10].

In 2019, there were 186 hospitals in Jakarta (135 general hospitals, 51 specialist hospitals), serving more than 10.5 million people across 661.5 km^2^ [11]. General hospitals with emergency departments (EDs) typically have their own ambulance fleet consisting of an average of two ambulances. Across Jakarta, only 85 ambulances belong to the government [12], and they are mainly used for patient transportation between hospitals rather than for emergency response [6] [13]. Overall, the number of ambulances in Jakarta is significantly lower than in high-income countries. For example, in Wales, there are 266 emergency ambulances (excluding other types of emergency vehicles) that serve a 3.1 million population and cover area of 4131 km^2^ [14]. This means, in Wales alone, the capacity of emergency ambulances is roughly 8.6 per 100,000 population, compared to 0.8 per 100,000 population in Jakarta. Indeed, across Asia Pacific countries, the variation in the number of ambulances ranges from 0.3 and 3.2 per 100,000 population [15].

Research has also documented different modes of transportation used by patients attending hospitals EDs. In high-income countries such as the United States, these include public transport, private car, air-medical transport, and multiple types of ambulance [16]. By contrast, in LMICs such as Bangladesh, transport modes include rickshaws, auto-rickshaws, and basic ambulances [17]. The utilization of ambulances by patients attending EDs varies and can be attributed to different factors such as socio-demographic characteristics and the type of emergency [16]. It is well established that patients with public health insurance (that covers the cost of using an ambulance) are more likely to use an ambulance [18], while poor ambulance performance (e.g., long response times) may have a negative effect on ambulance utilization. In Indonesia, there is currently no research on the utilization of ambulance services, primarily due to a lack of publicly available data or internationally accessible published literature.

This study aimed to investigate the use of ambulances by patients attending emergency departments in Jakarta, Indonesia. The primary objectives were to answer the following questions: 1) How do patients currently reach the hospital and does transportation mode differ by emergency type? 2) How do ambulances perform, in terms of measures such as response time or time to treatment, as compared with other modes of transport in Indonesia? As a secondary objective, we seek to understand the demand for ambulance services, from the perspective of the patient because a better understanding of spatial demand could enable the prioritisation of high-risk areas for improvement in the provision of pre-hospital and emergency services.

## Methodology

### Study design and setting

A survey was conducted in five major hospitals in Jakarta from December 1st to 31st 2019. These five hospitals comprised four government-funded and one private hospital. Two of the government hospitals accept nationwide referrals.

### Study sample

Patients who attended the hospital EDs during December 2019 were invited to take part in the study. Each patient had an equal chance to participate. Parents and guardians represented patients under 18 years of age. Patients or patients’ guardians also had the right to refuse involvement in the study. A total of 1964 out of 3179 patients (~ 62%) provided consent to participate. Reasons for refusing to participate were not recorded.

### Data collection

The questionnaire gathered information on the patients’ demographic profile, medical conditions, and the timing of the events involved in the patients’ journey to the emergency department. The questionnaire was adapted from Boutilier and Chan (2020) [17] who conducted a study of EMS in Dhaka, Bangladesh. Some modifications were made to suit the local context by local medical experts including the 118 Emergency Ambulance Director. The participating hospital’s ED directors reviewed and approved the questionnaire.

The hospitals normally collect data related to patients’ demographic profiles and medical conditions. However, the timestamp data related to the patient’s journey to the ED and the reasons for using their chosen transportation mode were not part of routine data collection. The journey time data was collected in the form of six ‘timestamps’; when the emergency happened, when the patient decided to go to the hospital and called for the transport, when transport arrived at the scene, when the patient departed to the hospital, when the patient arrived at the hospital, and when the patient received treatment for the first time. Based on these six timestamps, we defined the time duration for measuring the transport modes’ performance and time to treatment (stratified by different medical conditions). Similar analyses have been used in studying EMS in developed and developing countries [19]. We define the following six time intervals:

- *Patient delay*: the time between the emergency occurring and the decision to go to the hospital.
- *Response time:* the time between the decision to go to the hospital and transport arrival.
- *Time on the scene*: the time between the arrival of transport and departure to the hospital.
- *Travel time:* the time from leaving the scene until arrival at the hospital.
- *Patient waiting time:* the time from patient arrival at the hospital until the start of treatment.
- *Time to treatment*: the time duration between when the emergency occurred and when the patient received the treatment for the first time.

All data were collected by a hospital nurse using a paper-based questionnaire. EMS in Indonesia does not have a unifying triage protocol, so to reduce variation in data recording between hospitals, each hospital was provided with a manual on data types, a list of standard medical codes, and a formatted excel spreadsheet for recording the data. The study used a list of medical codes provided by the Welsh Ambulance Services in the UK (one of the partners in this study). The 118 Ambulance Services (also a partner in this study from the Indonesian side) appointed three staff members who were responsible for supervising the data collection. The routine data (e.g., demographics) obtained from the hospital were manually validated against the paper-based questionnaire by the study team.

### Data analysis

Data cleaning and analysis were conducted using open-source software R version 3.6.3 [20] (package tidyverse [21] and lubridate [22]). The normality tests were conducted (using the Shapiro-Wilk test) for each numerical variable at an aggregated level and group level, with a significant *p*-value < 0.05. Based on the results, comparisons between groups were conducted using the Kruskal-Wallis test and the result was considered significant if the *p*-value < 0.05. Continuous data such as age and duration were reported using median and IQR. Categorical data, such as types of transportation modes and medical conditions were reported using percentages and counts. Results from a preliminary data analysis were presented and discussed with the local medical experts involved in the study.

## Results

### Basic demographic characteristics

The total number of patients who participated in the survey was 1964 out of 3179 (62%), of which 1051 (51.7%) were male and 949 (48.3%) were female. These figures are close to the proportion of the 2019 population estimate for males and females in Jakarta at 50.4 and 49.6%, respectively [11]. The median age for all patients was 44 years old (IQR: 26 to 58 years). Note that 75% of the population in Jakarta is between 0 and 44 years of age [9]. Around 50.3% of patients were not currently employed or retired, and of those who reported income, 35.6% earned between 1 and 3 million Indonesian Rupiah (IDR), equivalent to £55.46 – £166.37 per month.

### Medical conditions

The analysis revealed 153 unique chief complaints with the five highest occurrences being pyrexia (7.8%), dyspnoea (6.2%), hypertension (4.5%), diabetes (4.2%), and lower respiratory tract infection (3.8%). For the purpose of analysis and reporting, we used the categorisation of medical codes: Medical, Respiratory, Trauma, Cardiovascular, and Neurological. We grouped problems related to obstetrics, paediatrics, self-harm, and mental health into “Other”, due to small occurrences (2.9% combined). Category “Cardiovascular” included both general cardiovascular and acute coronary syndrome. Similar classifications have been used in a study concerning ED attendance in an Indonesian hospital [23]. Table 1 presents a summary of the medical conditions by category and the corresponding age of patients. The majority of patients attending the emergency department had general medical problems (63%).

**Table 1:** Summary of patient’s age (years) by medical groups

Medical Group	Total		Female		Male	
	N (%)	Median age (IQR)	N (%)	Median age (IQR)	N (%)	Median age (IQR)
Medical	1236 (62.9)	41.5 (24,57)	637 (67.1)	41 (25,56)	599 (59.0)	42 (23,58)
Respiratory	301 (15.3)	47 (30,59)	126 (13.3)	50 (29.2,59)	175 (17.2)	47 (31.5,59.2)
Trauma	169 (8.6)	35 (23,51)	60 (6.3)	35.5 (22.5,55.2)	109 (10.7)	33 (24,48)
Cardiovascular	130 (6.6)	55 (47,63)	50 (5.3)	57.5 (48.2,71.2)	80 (7.9)	54 (47,59.2)
Neurological	72 (3.7)	59 (50.8,66.2)	37 (3.9)	63 (56,69)	35 (3.4)	55 (48,61.5)
Other	56 (2.9)	29 (22,35.2)	39 (4.1)	29 (23,35)	17 (1.7)	29 (8,55)
Total	1964 (100)	44 (26,58)	949 (100)	44 (26,58)	1015 (100)	44 (26,58)

### Transportation modes

Table 2 summarises the transportation modes used by patients and the reasons related to the choice of transportation. The results from analysing transport mode showed that ‘Own Car’ and ‘Ride-sharing service car’ categories were the most used transportation modes at 30.3 and 30.4%, respectively. Ambulances were used by 9.3% of patients, while 19.7% of patients used motorcycles to reach hospitals. Public transport and taxi shared similar proportions at 3.3 and 3.1%, respectively. The category “Other” (3.9%) included CNG-fuelled three-wheeler vehicles.

**Table 2:** Transport choices, reasons, and costs

Transport mode:	Number	Percentage (%)
Ride-sharing service car	598	30.4
Own car	596	30.3
Motorcycle	387	19.7
Ambulance	182	9.3
Other	75	3.8
Taxi	61	3.1
Reasons for using ambulance:	Number	Percentage (%)
Affordable	13	7.2
Medical condition	19	50.3
Advice from doctor/others	42	23.2
Own initiative	26	14.4
Other	9	5.0
Reasons for not using ambulance:	Number	Percentage (%)
Too expensive	139	7.8
Not available	217	12.2
Takes too long	315	17.7
Not necessary	370	20.8
Not aware	675	37.9
Other	67	3.8
Do the patients know how to contact an ambulance?	Number	Percentage (%)
Yes	480	24.4
No	1475	75.1
NA	9	0.5
Reasons for choosing the hospital:	Number	Percentage (%)
Nearest hospital	747	38.0
Referral hospital	398	20.3
Been treated before	311	15.8
Inexpensive	19	1.0
Government hospital	337	17.2
Personal reason	111	5.7
Other	42	2.1
Transport cost (IDR (GBP))^a^	Number	Percentage (%)
< 100 k (< £5.54)	1585	80.7
100–500 k (£5.54 - £27.68)	247	12.6
500–1000 k (£27.68 - £55.37)	89	4.5
> 1000 k (> £55.37)	23	1.2
Unknown	20	1.0

^a^The conversion rate Rp18061.29 to £1 was obtained from xe.com on 20/05/2020

All figures are rounded to 1 decimal point. The summation of the percentages may exceed 100% due to the rounding effect

The majority of patients who used an ambulance reported doing so because their medical conditions were considered severe and required emergency attention (50.3%). The next most common reason was medical advice (23.2%). Only 7.2% of patients who used an ambulance thought that it was affordable.

Many patients did not use an ambulance because they were not aware of ambulance services (37.9%). A proportion of patients thought that it was not necessary to call the ambulance (20.8%), whereas others said that the ambulance took too long to arrive (17.7%). Some patients tried to contact an ambulance service, but it was not available (12.2%). Only 7.8% of patients who did not use an ambulance thought the ambulance was expensive. More than 75% of patients did not know how to contact an ambulance (and this included patients who ultimately did and did not use an ambulance). There are currently six emergency numbers operating in Indonesia, which may cause confusion in the event of an emergency. Patients who did not know how to contact an ambulance but ended up using an ambulance might have received help from a bystander or family member.

The costs associated with transport to hospital varied. The majority (80.7%) of patients reported that transportation costs were less than £6, and 96.7% of these patients represented non-ambulance users. Among the patients who used an ambulance, over 65.4% spent between £6 and £55, while 5% spent over £55. Overall, these results indicate that using an ambulance was more costly compared to other transport modes.

Around 46.2% of the ambulance users came to the hospitals by referral, compared to 17.6% of patients who did not use the ambulance. Roughly 40% of patients who did not use ambulances, chose the nearest hospitals to receive treatment compared to 19.2% of ambulance users.

### Medical conditions versus transportation modes

**Fig. 1:**
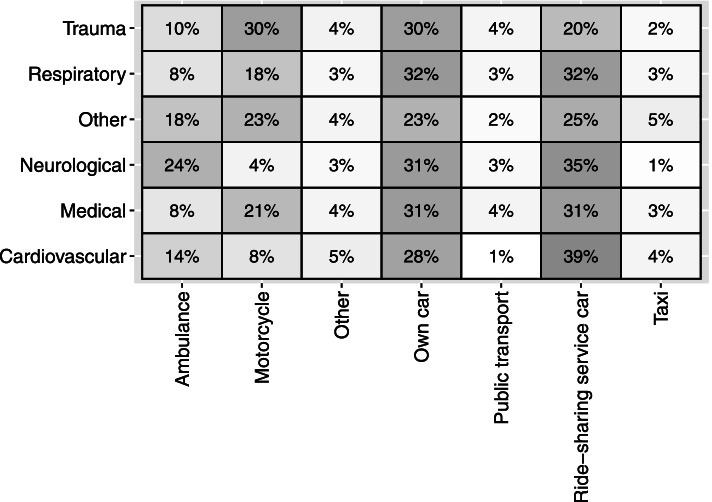
Heat map showing the distribution of transport choices and medical categories

Figure 1 shows a heat map describing the percentage of patients, grouped by each medical category, who attended a hospital with different transportation modes. Across all medical conditions, the majority of patients used either their own car or a ride-sharing service car. Ambulances were often the third or fourth choice for transportation, including for those patients with life-threatening conditions. A large proportion of patients with trauma problems (30%) used motorcycles to reach the hospital, while only 10% of patients with trauma and 14% with cardiovascular diseases used an ambulance. The majority of trauma patients who did not arrive via an ambulance stated that they did not know that ambulance services exist (35.5%), that it was too long to wait for an ambulance (25.7%), or that an ambulance was not available when contacted (9.9%).

A more detailed breakdown of chief complaints under the “Medical” categorisation and the associated means of transport is given in the heat map shown in Supplementary Fig. 2, Additional file 1. At an aggregated level, pyrexia made up the largest group of patients (12.4%). Patients with this illness came to the hospital by many different modes of transport, including motorcycles (28.8%) and their own car (34.0%). Patients who used an ambulance to go to the hospital were mainly those with diabetes (9.3%) and hypertension problems (6.0%).

### Timestamp analysis

Supplementary Tables 3 and 4, in Additional files 2 and 3, provide details on the six time intervals separated by transport mode and medical category, respectively. In the following sub-sections, we highlight details for each time interval.

#### Patient delay

Overall, the median duration of patient delay was 24 minutes with an IQR ranging from 6 to 60 minutes. Across different types of transport mode, the median patient delay ranged from 18 to 30 minutes. The median duration of patient delay was significantly different between medical groups. Patients with respiratory and neurological problems had similar median delays of 30 minutes, which was 6 minutes longer than average. In contrast, 75% of patients with cardiovascular diseases and respiratory problems took up to 2 hours to decide to go to the hospital. Patients’ lack of awareness of the symptoms may have contributed to the delay in making a decision.

#### Response time

Our results indicate that 75% of response times were more than 30 minutes. The median response time for an ambulance was 24 minutes with an IQR from 12 to 54 minutes, which was the longest compared to all other modes of transport. Motorcycles had the shortest median response time of 6 minutes with an IQR ranging from 0 to 18 minutes. The high IQR for ambulances is likely at least in part because ambulances have the furthest distance to travel, and they do not have an automatic right to priority in congested traffic. Patients experiencing cardiovascular diseases had a similar median duration of transport response as those with neurological and respiratory problems (18 minutes). The shortest duration was experienced by those patients with trauma injuries (median 6 minutes, IQR: 6 to 18 minutes).

#### Time on scene

At the aggregated level, the median time spent on the scene by all modes of transport was 0.3 minutes (IQR from 0 to 10 minutes). For ambulances, the median time on the scene was 0.5 minutes, and 75% of ambulances spent less than 15 minutes on the scene. There was no significant difference in time spent on the scene for different medical conditions. The relatively short duration of time on scene is likely because the majority of transport modes were patients’ own cars and motorcycles which were (likely) readily available and did not involve any treatment. Similarly, the short duration for ambulances may be because patients were referred from other healthcare facilities where an ambulance was available and treatment was not needed. Indeed, we found that patients who used ambulances were referral patients (46.2%), but we did not record the patients’ origin prior to arriving at the hospital ED.

#### Travel time (to hospital)

The duration of travel to the hospital showed a significant difference among at least two different transport modes. Ambulance and own car shared a similar median of 42 minutes (IQR: 30 to 60 minutes), while the shortest median travel time was experienced by patients using motorcycles (30 minutes, IQR: 18 to 42 minutes). There was no significant time difference in the duration of travel for different medical conditions.

#### Patient waiting time

The results for the patient waiting time by different transport modes did not show any significant difference. However, (as expected) different medical conditions showed differences in the time patients spent waiting to be treated. For example, patients with trauma, cardiovascular, and respiratory problems shared a similar median time of 5 minutes when waiting to be treated (IQR ranged from 0 to 10 minutes), which was slightly longer than general medical conditions. This might be explained by the fact that patients with trauma, cardiovascular, or respiratory problems will need to be seen by a doctor who may not be readily available, while patients with more general medical problems can be seen directly (and typically immediately) by a nurse.

#### Time to treatment

Patients who arrived via ambulance had the longest median time to treatment (120 minutes, IQR: 78 to 270 minutes). In terms of the medical condition, patients with neurological problems experienced the longest time to treatment (median 120 minutes, IQR: 84 to 276 minutes).

## Discussion

The goal of this study was to investigate the use of ambulances by patients attending emergency departments in Jakarta, Indonesia. Our primary objectives were to answer the following questions: 1) How do patients currently reach the hospital and does transportation mode differ by emergency type? 2) How do ambulances perform, in terms of measures such as response time or time to treatment, as compared with other modes of transport in Indonesia?

For the first question, we found that patients travelled to the hospital by various modes of transport and in many cases these modes were not suitable for their conditions. The majority of patients with trauma or respiratory problems used motorcycles or their own cars, and a large number of patients with cardiovascular diseases did not use an ambulance (as would be recommended) [24]. We found only 10% of patients with trauma travelled with an ambulance, which confirms previous findings, that in Pan Asian countries, less than 20% of trauma patients use ambulances [25]. Currently, ambulances in Indonesia are used mainly for referrals or inter hospitals transportation [13], rather than emergency response [1] so the low utilization of ambulances (9.3%) is not surprising. In reality, patients who are aware of the existence of the ambulance service prefer to prioritise rapid access to professional treatment at the hospital rather than waiting the additional time for an ambulance to arrive. Ambulance utilization in our study was lower compared to the results found in another study within Indonesia, which determined that 36.7% of ED patients used ambulances [23]. This difference might be explained by the fact that the study was conducted in another region and only involved 139 patients.

A key factor that may influence the low utilization of ambulances is the high cost associated with the service. The lack of comprehensive coverage of ambulance costs in health insurance is a known barrier to access to emergency transport in developing countries [26]. In 2014, Indonesia introduced universal health care which provides equal access for all citizens [27]. However, only ambulances provided by the government are covered by the universal health care policy. Unfortunately, the service is compromised by long waiting times and although each healthcare facility with an ED has its own ambulance fleet, there is no regulation or standardisation concerning the provision of emergency care. This contributes to variation in the services, including fees charged for the services, which may erode patient confidence. A possible strategy that may alleviate this issue is to integrate all ambulance services into the existing healthcare system, tailored to local needs and conditions [28]. The inclusion of emergency transport (private and public) within the national health insurance may serve as a solution because this would enable equal access for individuals in need and minimise the impact on healthcare costs.

For the second question, we found that ambulances exhibited longer response times (median 24 minutes, IQR: 12 to 54 minutes) as compared to other transport modes (median 12 minutes, IQR: 0 to 30 minutes). According to local experts involved in the current study, ambulance services in Jakarta have a response time target of 10 minutes for life-threatening conditions. However, it is difficult to determine how often this target is met due to the lack of publicly available published reports. In neighbouring countries such as Singapore and Malaysia, median ambulance response times were reported at 11.4 minutes (SD: 4.9 minutes) and 15.2 minutes (SD: 6.7 minutes), respectively [29, 30]. In the UK, the national target for ambulance response time is 8 minutes for life-threatening conditions and 19 minutes for serious but not immediately life-threatening conditions [31]. Even with significantly more resources (in the UK) as compared to Indonesia, achieving the response target at all times is challenging [31, 32]. External factors such as patient’s location relative to ambulance posts, weather, traffic, or road conditions may also influence the ambulance response time [29, 33]. In Jakarta, there is a lack of priority given to ambulance vehicles due to congested traffic, which means that ambulances travel no faster than ordinary traffic, creating a situation where an ambulance is (almost) always likely to be the slowest form of transport to a hospital. Future policy research should focus on educating the public about the importance of yielding for ambulances.

The majority of patients attending hospital emergency departments had general medical problems (63%), and only 15.2% reported life-threatening conditions such as trauma and cardiovascular diseases. Similar results were found in a study on ED attendance in an Indonesian hospital; using 2015 data, roughly 70% of patients were categorised as non-trauma cases [34]. Studies have found that a significant number of patients attending hospital emergency departments had non-emergency conditions [35]. However, this does not suggest that the patients did not have serious underlying conditions [36]. Patients’ perceived illness severity and the accessibility of the emergency department compared to other clinics may have motivated patients to attend the hospital emergency department [37]. The low number of patients with life-threatening conditions may also be because patients with such conditions will access specialist hospitals in Jakarta (that were not included in our survey).

In the specific context of Indonesia, patients with a life-threatening condition such as acute coronary syndrome (ACS) often seek treatment at home first and only consult a hospital when their conditions deteriorate [24]. Delay in contacting the hospital often leads to delays in receiving treatment from a healthcare professional. Indeed, we found that patients with trauma and cardiovascular diseases spent up to 5 hours between the onset of symptoms and receiving treatment. This long time to treatment appears common for acute myocardial infarction in developing countries [38], which is alarming since a delay in receiving pre-hospital care for patients with these conditions can increase hospital mortality [39, 40, 41]. Patients’ lack of awareness of the symptoms can contribute to the delay in accessing pre-hospital and emergency services [42]. To reduce the delay, healthcare providers and policymakers should focus on improving patients’ awareness of related symptoms, especially for myocardial infarction.

### Limitations and future study

This study is subject to some potential limitations and biases that warrant careful interpretation of the generalisability of the results. First, not all patients who attended the hospital EDs consented to be involved in the survey, and reasons for not wanting to be involved were not recorded to avoid inconvenience and embarrassment. If a large number of patients with severe conditions were among those who did not participate, this could impact the results. Second, the time data was dependent on patients’ recall, which may not be entirely accurate. Generally, time data, in relation to transportation mode used by the patients, is not part of hospital data collection. Including this time data, wherever appropriate, in routine hospital data collection could be introduced in the future so that stakeholders can better evaluate the effectiveness and efficiency of healthcare services. Third, we were not able to follow up on patient outcomes. It would be useful to measure patient outcomes based on different transportation modes and the corresponding time to treatment. Finally, the survey involved a relatively small number of hospitals, even though care was taken to try and work with a representative sample of hospitals with emergency departments. Future studies may include more hospitals across Jakarta and possibly combine administrative data and survey data. The administrative data can be used to validate and give a more accurate picture of a patient’s medical conditions and the outcomes of the receiving treatment.

Our study was conducted due to the scarcity of publicly available reports or data concerning emergency medical service utilization in Indonesia. We contribute to the literature concerning the utilization of emergency services in Jakarta-Indonesia, particularly from the perspective of patients. We do not claim the generalisability of the results beyond the sample population, as a different population in a different region of Indonesia may have different characteristics. We suggest more studies should be done in the future in different regions, covering both urban and suburban areas in Indonesia. We also stress the need for future studies that collect data directly from ambulance providers in Jakarta (or other areas of Indonesia). This will enable a more granular study of emergency healthcare demand and available capacity using tools such as computer simulation. Such a study may provide a better picture of the current utilization of the emergency services, and inform policy decisions related to the impact of healthcare resource allocation decisions.

## Conclusions

The study found that the low utilization of emergency ambulance services in Jakarta could be related to a range of factors including the ability or willingness to pay for the service, lack of awareness of symptoms that need emergency transportation, lack of awareness of the existence of the ambulance services, and disinclination to use an ambulance due to long response times.

## Supplementary Information

**Additional file 1: Supplementary Figure 2.** Heat map; Distribution of transport choices and medical conditions.

**Additional file 2: Supplementary Table 3.** Summary for the time analysis by different transportation modes used by the patients. Med = median, IQR = (Q1, Q3).

**Additional file 3: Supplementary Table 4.** Summary for the time analysis by different medical groups of main health problems experienced by the patients. Med = median, IQR = (Q1, Q3).

## Data Availability

Information on the data underpinning the results presented here, including how to access them, can be found in the Cardiff University data catalogue at 10.17035/d.2022.0177980501.
