# Supplementary material for: Emergency services utilization in Jakarta (Indonesia): a cross-sectional study of patients attending hospital emergency departments
Source: BMC Health Serv Res. 2022 May 13;22:639. doi: 10.1186/s12913-022-08061-8 (PMC9103083; doi:10.1186/s12913-022-08061-8)
Supplement: Supplementary file 1 — Additional file 1: Supplementary Figure 2. Heat map; Distribution of transport choices and medical conditions. [file 12913_2022_8061_MOESM1_ESM.pdf]

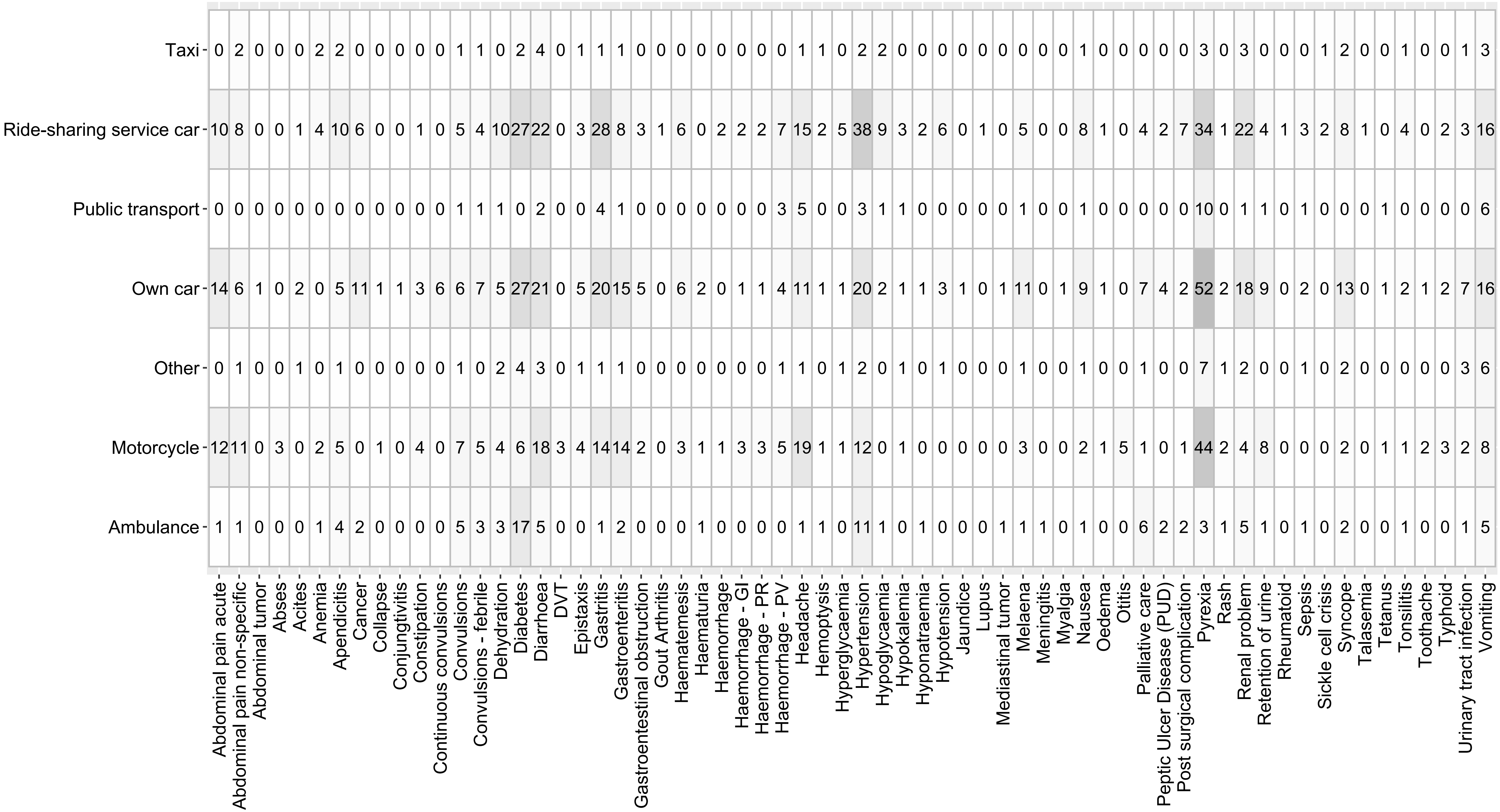
Taxi 0 2 0 0 0 2 2 0 0 0 0 0 1 1 0 2 4 0 1 1 1 0 0 0 0 0 0 0 0 1 1 0 2 2 0 0 0 0 0 0 0 0 0 1 0 0 0 0 0 3 0 3 0 0 0 1 2 0 0 1 0 0 1 3
Ride-sharing service car 10 8 0 0 1 4 10 6 0 0 1 0 5 4 10 27 22 0 3 28 8 3 1 6 0 2 2 2 7 15 2 5 38 9 3 2 6 0 1 0 5 0 0 8 1 0 4 2 7 34 1 22 4 1 3 2 8 1 0 4 0 2 3 16
Public transport 0 0 0 0 0 0 0 0 0 0 0 0 1 1 1 0 2 0 0 4 1 0 0 0 0 0 0 0 3 5 0 0 3 1 1 0 0 0 0 0 1 0 0 1 0 0 0 0 0 10 0 1 1 0 1 0 0 0 1 0 0 0 0 6
Own car 14 6 1 0 2 0 5 11 1 1 3 6 6 7 5 27 21 0 5 20 15 5 0 6 2 0 1 1 4 11 1 1 20 2 1 1 3 1 0 1 11 0 1 9 1 0 7 4 2 52 2 18 9 0 2 0 13 0 1 2 1 2 7 16
Other 0 1 0 0 1 0 1 0 0 0 0 0 1 0 2 4 3 0 1 1 1 0 0 0 0 0 0 0 1 1 0 1 2 0 1 0 1 0 0 0 1 0 0 1 0 0 1 0 0 7 1 2 0 0 1 0 2 0 0 0 0 0 3 6
Motorcycle 12 11 0 3 0 2 5 0 1 0 4 0 7 5 4 6 18 3 4 14 14 2 0 3 1 1 3 3 5 19 1 1 12 0 1 0 0 0 0 0 3 0 0 2 1 5 1 0 1 44 2 4 8 0 0 0 2 0 1 1 2 3 2 8
Ambulance 1 1 0 0 0 1 4 2 0 0 0 0 5 3 3 17 5 0 0 1 2 0 0 0 1 0 0 0 0 1 1 0 11 1 0 1 0 0 0 1 1 1 0 1 0 0 6 2 2 3 1 5 1 0 1 0 2 0 0 1 0 0 1 5
Abdominal pain acute
Abdominal pain non-specific
Abdominal tumor
Abses
Acites
Anemia
Apendicitis
Cancer
Collapse
Conjungtivitis
Constipation
Continuous convulsions
Convulsions
Convulsions - febrile
Dehydration
Diabetes
Diarrhoea
DVT
Epistaxis
Gastritis
Gastroenteritis
Gastroentestinal obstruction
Gout Arthritis
Haematemesis
Haematuria
Haemorrhage
Haemorrhage - GI
Haemorrhage - PR
Haemorrhage - PV
Headache
Hemoptysis
Hyperglycaemia
Hypertension
Hypoglycaemia
Hypokalemia
Hyponatraemia
Hypotension
Jaundice
Lupus
Mediastinal tumor
Melaena
Meningitis
Myalgia
Nausea
Oedema
Otitis
Palliative care
Peptic Ulcer Disease (PUD)
Post surgical complication
Pyrexia
Rash
Renal problem
Retention of urine
Rheumatoid
Sepsis
Sickle cell crisis
Syncope
Talasemia
Tetanus
Tonsilitis
Toothache
Typhoid
Urinary tract infection
Vomiting
