# Supplementary material for: Emergency services utilization in Jakarta (Indonesia): a cross-sectional study of patients attending hospital emergency departments
Source: BMC Health Serv Res. 2022 May 13;22:639. doi: 10.1186/s12913-022-08061-8 (PMC9103083; doi:10.1186/s12913-022-08061-8)
Supplement: Supplementary file 2 — Additional file 2: Supplementary Table 3. Summary for the time analysis by different transportation modes used by the patients. Med = median, IQR = (Q1, Q3). [file 12913_2022_8061_MOESM2_ESM.docx]

*Supplementary Table 3. Summary for the time analysis by different transportation modes used by the patients. Med = median, IQR = (Q1, Q3)*

| Time analysis | Combined  (N = 1965) | Ambulance  (N = 182) | Own car  (N = 596) | Ride-sharing service car  (N = 598) | Motorcycle  (N = 387) | Taxi  (N = 61) | Public transport  (N = 65) | Other  (N = 75) |
| --- | --- | --- | --- | --- | --- | --- | --- | --- |
|  | Med (IQR) | Med (IQR) | Med (IQR) | Med (IQR) | Med (IQR) | Med (IQR) | Med (IQR) | Med (IQR) |
| Patient delays (hours) | 0.4 (0.1 , 1.0) | 0.3 (0.1 , 1.0) | 0.3 (0.1 , 1.0) | 0.5 (0.2 , 1.0) | 0.5 (0.1 , 1.2) | 0.3 (0.1 , 1.0) | 0.3 (0.2 , 1.5) | 0.5 (0.1 , 1.5) |
| Transport response time (hours) | 0.2 (0.0 , 0.5) | 0.4 (0.2 , 0.9) | 0.2 (0.0 , 0.5) | 0.3 (0.2 , 0.5) | 0.1 (0.0 , 0.3) | 0.3 (0.2 , 0.4) | 0.3 (0.1 , 0.5) | 0.2 (0.0 , 0.5) |
| Time on scene (minutes) | 0.3 (0.0 , 10.0) | 5.0 (0.0 , 15.0) | 2.5 (0.0 , 10.0) | 5.0 (0.0 , 10.0) | 0.0 (0.0 , 10.0) | 0.0 (0.0 , 5.0) | 5.0 (0.0 , 15.0) | 0.0 (0.0 , 0.0) |
| Travel time (hours) | 0.6 (0.4 , 0.9) | 0.7 (0.5 , 1.0) | 0.7 (0.5 , 1.0) | 0.6 (0.4 , 0.9) | 0.5 (0.3 , 0.7) | 0.6 (0.5 , 0.8) | 0.7 (0.5 , 0.9) | 0.6 (0.3 , 1.0) |
| Patient waiting time (minutes) | 5.0 (0.0 , 10.0) | 0.0 (0.0 , 10.0) | 4.5 (0.0 , 10,0) | 3.0 (0.0 , 10.0) | 5.0 (0.0 , 10.0) | 3.0 (0.0 , 14.0) | 5.0 (0.0 , 10.0) | 5.0 (0.0 , 14.5) |
| Time to treatment (hours) | 1.8 (1.1 , 3.0) | 2.0 (1.3 , 4.5) | 1.7 (1.1 , 3.0) | 1.9 (1.2 , 3.1) | 1.5 (1.0 , 2.5) | 1.5 (1.1 , 2.5) | 2.0 (1.3 , 3.4) | 2.0 (1.1 , 3.4) |
