# Supplementary material for: Emergency services utilization in Jakarta (Indonesia): a cross-sectional study of patients attending hospital emergency departments
Source: BMC Health Serv Res. 2022 May 13;22:639. doi: 10.1186/s12913-022-08061-8 (PMC9103083; doi:10.1186/s12913-022-08061-8)
Supplement: Supplementary file 3 — Additional file 3: Supplementary Table 4. Summary for the time analysis by different medical groups of main health problems experienced by the patients. Med = median, IQR = (Q1, Q3). [file 12913_2022_8061_MOESM3_ESM.docx]

*Supplementary Table 4. Summary for the time analysis by different medical groups of main health problems experienced by the patients. Med = median, IQR = (Q1, Q3)*

| Time analysis | Combined  (N = 1964) | General Medical  (N = 1236) | Cardiovascular  (N = 130) | Respiratory  (N = 301) | Trauma  (N = 169) | | Neurological  (N = 72) | Other  (N = 56) |
| --- | --- | --- | --- | --- | --- | --- | --- | --- |
|  | Med (IQR) | Med (IQR) | Med (IQR) | Med (IQR) | | Mead (IQR) | Med (IQR) | Med (IQR) |
| Patient delays (hours) | 0.4 (0.1 , 1.0) | 0.3 (0.2 , 1.0) | 0.3 (0.1 , 2.0) | 0.5 (0.2 , 2.0) | | 0.3 (0.1 , 1.0) | 0.5 (0.1 , 1.5) | 0.5 (0.2 , 1.0) |
| Transport response time (hours) | 0.2 (0.0 , 0.5) | 0.2 (0.0 , 0.5) | 0.3 (0.1 , 0.5) | 0.3 (0.1 , 0.5) | | 0.1 (0.0 , 0.3) | 0.3 (0.0 , 0.6) | 0.2 (0.0 , 0.3) |
| Time on scene (minutes) | 0.3 (0.0 , 10.0) | 2.0 (0.0 , 10.0) | 0.0 (0.0 , 5.0) | 0.0 (0.0 , 10.0) | | 0.0 (0.0 , 10.0) | 0.0 (0.0 , 11.3) | 5.0 (0.0 , 10.0) |
| Travel time (hours) | 0.6 (0.4 , 0.9) | 0.6 (0.4 , 0.9) | 0.5 (0.4 , 0.9) | 0.6 (0.4 , 0.9) | | 0.6 (0.3 , 0.8) | 0.7 (0.4 , 1.1) | 0.6 (0.4 , 0.9) |
| Patient waiting time (minutes) | 5.0 (0.0 , 10.0) | 3.0 (0.0 , 10.0) | 5.0 (0.0 , 10.0) | 5.0 (0.0 , 10.0) | | 5.0 (0.0 , 10.0) | 0.0 (0.0 , 5.0) | 5.0 (0.0 , 10.0) |
| Time to treatment (hours) | 1.8 (1.1 , 3.0) | 1.8 (1.1 , 2.8) | 1.7 (1.1 , 5.0) | 2.0 (1.2 , 3.8) | | 1.3 (1.0 , 2.4) | 2.0 (1.4 , 4.6) | 1.5 (1.1 , 3.0) |
